# Improved Degradome Sequencing Protocol via Reagent Recycling from sRNAseq Library Preparations

**DOI:** 10.3390/ijms26147020

**Published:** 2025-07-21

**Authors:** Marta Puchta-Jasińska, Jolanta Groszyk, Maja Boczkowska

**Affiliations:** Plant Breeding and Acclimatization Institute—National Research Institute, 05-870 Radzików, Poland; j.groszyk@ihar.edu.pl (J.G.); m.boczkowska@ihar.edu.pl (M.B.)

**Keywords:** degradome, cost-effective, protocol, miRNA, target genes, barley seeds

## Abstract

One of the key elements in the analysis of gene expression and its post-translational regulation is miRNAs. Degradome-seq analyses are performed to analyze the cleavage of target RNAs in the transcriptome. This work presents the first degradome-seq library preparation protocol that enables successful construction of libraries, even from highly degraded RNA samples with RIN below 3, thus significantly expanding the possibilities for research when working with low-quality material. The developed protocol improves the efficiency of library preparation in degradome-seq analysis used to identify miRNA targets, reduces library preparation time, and lowers the cost of purchasing reagents by using reagents from the RNA-seq library preparation kit and proprietary-designed primers. A crucial feature of this new protocol is optimizing the purification step for short library fragments, which increases the yield of correctly sized fragments compared to previously used methods. This is achieved by implementing an original method involving tube-spin purification with gauze and precipitation using sodium acetate with glycogen, greatly enhancing recovery efficiency—a factor especially critical when working with degraded RNA. Cloning to a plasmid and sequencing of the inserted fragment verified the correctness of the library preparation using the developed protocol. This protocol represents a groundbreaking tool for degradome research, enabling the construction and sequencing of degradome libraries, even from degraded samples previously considered unsuitable for such analyses. This is due to the use of residues from the sRNA-seq library kit. It noticeably reduces the cost of library construction. The precision of the excised fragment after electrophoresis was performed during the procedure to isolate fragments of the correct length, which was improved using additional size markers. Compared to previously used methods, optimizing the purification method of degradome-seq libraries allowed an increase in the yield of fragments obtained.

## 1. Introduction

In plants, microRNAs (miRNAs) primarily function by cleaving target messenger RNA (mRNA) molecules, which have near-perfect complementarity at the matching sites [1]. Computational methods based on genome-wide searches for targets for miRNAs, which rely on base pairing and assessment of miRNA–target interactions, have yielded a high percentage of false-positive predictions [2,3]. Pairing miRNAs with target genes is a complex process influenced by numerous factors, including expression patterns over time. Therefore, experimental verification of targets is a crucial step in the research process [4]. In 2008, three methods for the verification of miRNA targets based on next-generation sequencing were published, i.e., parallel analysis of RNA ends (PARE) [5], degradome sequencing (degradome-seq) [6], and genome-wide mapping of uncapped and cleaved transcripts (GMUCT) [7]. The methods above rely on capturing a sequence devoid of a cap at the 5′ side of a cut 3′ mRNA that has been ligated with an adapter [6]. Among them, degradome-seq represents a high-throughput sequencing method that draws inspiration from a modified 5′-rapid amplification of cDNA ends (5′-RACE) approach [8]. Degradome sequencing enables the identification of target genes subjected to cleavage by miRNAs. The methodology presented here constitutes a modification of the one initially described by Lin et al. (2019) [9]. This method facilitates the preparation of libraries in a more streamlined and efficient manner while also enabling the utilization of residual components from the NebNext small RNA Library Prep Set for Illumina (New England Biolabs) following the preparation of miRNA sequencing libraries. Degradome profiles can provide substantial insight into the intricacies of RNA processing [9,10]. The method generates a fixed fragment length library through enzymatic restriction using *Mme I*, which cleaves 20 bp 3′ of the recognition site included in the 5′ cDNA adapter. Subsequently, 3′ dsDNA adapters are ligated to the digestion products, and the fragments are then subjected to PCR amplification, purification, and appropriate sequencing [1]. A significant difficulty in the degradome-seq library construction methods described so far is the isolation of relatively large amounts of RNA (about 5 µg) with a RIN value above 7. Obtaining such high-quality RNA in plants or biobanked samples could be challenging [11]. Traditional protocols require multiple library construction steps, each requiring specialized reagents, substantially increasing the cost of library preparation [12,13]. Another difficulty lies in precisely purifying specific cDNA fragments corresponding to transcripts cut by miRNAs, which requires high-resolution size selection. This step often involves repetition and the loss of a large amount of material, making it challenging to achieve high-throughput library construction and high-quality results. To date, using polyacrylamide gels for purification results in a maximum fragment recovery of 30% [14].

Analyzing networks regulating miRNA-mediated gene expression based on degradome sequencing data presents a superior approach to miRNA targets’ computational (in silico) prediction. Additionally, interactions between small interfering RNA (siRNAs) and gene expression alterations can be monitored using degradome sequencing data [15,16,17]. Recently, studies have also demonstrated the potential of such data in RNA research. For example, it can be used to map endoribonucleolytic cleavage sites in vivo, identify conserved motifs at the ends of 5′ naked RNA fragments, and search for regions associated with stacked ribosomes (or other RNA-binding proteins) on transcripts [15]. The rate of false-positive predictions of miRNA binding sites and the search space size for miRNA target sites were significantly reduced by applying CLIP-Seq and degradome-seq methods. Identifying and validating their targets is crucial to elucidating the biological function of small regulatory RNAs (sRNAs). Most computational tools for predicting sRNA targets in plants (and animals) employ techniques that seek to identify complementarity between an sRNA sequence and a potential target sequence [18,19,20].

This study aimed to develop and validate a new protocol for efficiently preparing degradome libraries, even from degraded RNA samples, using reagents remaining after sRNAseq library preparation [21].

## 2. Results and Discussion

**Figure 1:**
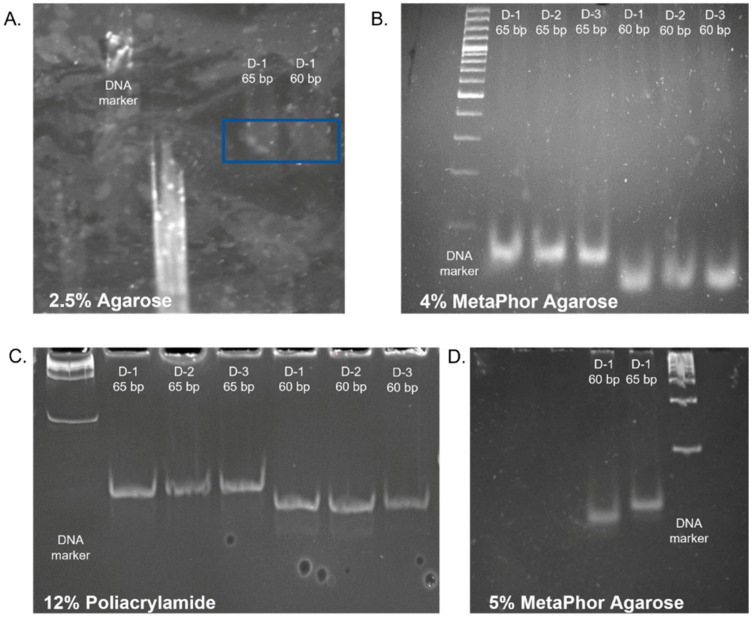
A qualitative assessment of the separation of 60, 65 bp samples, and the separation was performed in: (**A**) 2.5% agarose gel (samples were mark by box); (**B**) 4% MetaPhor™ high-resolution agarose, Lonza; (**C**) 12% polyacrylamide gel; (**D**) 5% high-resolution MetaPhor™ agarose (D-1; D-2; D-3-technical replicates).

Several optimizations and verifications were performed to prepare the protocol. The protocol has two notable innovations. Firstly, it allows for more efficient extraction of libraries before the binding of Illumina adapters, thereby enhancing the overall efficiency of the method. Secondly, it employs automated electrophoresis as the final purification technique for libraries, which ensures superior quality of the final library. The degradome assay is vital for gaining insights into the sensitivity of RNA decay pathways to differential specialization. Previously standard degradome methods based on PARE and GMUCT required complicated steps involving RNA binding, purification, and size selection [22]. The modified 5P-seq method involves multi-step enzymatic reactions and purification with magnetic beads, which can lead to high reagent costs and material losses. The 5P-seq method requires DSN-mediated rRNA depletion at the initial stage [22]. So, libraries can be prepared quickly (in as little as 9 h) with the proposed HT-5P-seq modification. However, an effort still needs to be made to build rRNA capture probes and supply large quantities (around 5 µg) of RNA input material [22]. A table in Supplementary Materials S4 summarizes the main features of the protocols mentioned above, including RNA quality requirements, input amounts, library preparation time, cost, fragment recovery efficiency, adaptability to degraded RNA, and platform compatibility. The article’s proposed method involves reducing the number of enzymatic reactions and omitting probes and ribodepletions. Our method uses poly(A)-binding mRNA capture based on RNA-seq library construction reagents. Tests were conducted to identify the most effective methodology for visualizing and extracting libraries from gels to enhance the overall library construction process. The fragments must be 60–65 bp in length, precluding purification of the samples via both automatic electrophoresis and magnetic beads. The components and conditions of the individual steps in the preparation of the libraries have been optimized for challenging materials, such as grains of long-term stored cereals that are rich in reserve substances, e.g., sugars. To this end, primers designed for the PCR-amplified libraries were purified using 4% MetaPhor™ high-resolution agarose gels (Lonza). Custom-size markers of 60 and 65 bp, generated by amplifying an ADP-ribosylation factor fragment, facilitated precise excision of the target DNA smear. DNA fragments were subsequently recovered by centrifugation through gauze-lined tubes, followed by ethanol precipitation in the presence of sodium acetate and glycogen. For comparative evaluation, alternative purification methods—including polyacrylamide gel extraction and commercially available purification kits—were also assessed (see Supplementary Materials S3). The quality and size distribution of the final libraries were confirmed using an Agilent 2100 Bioanalyzer, and library concentrations were determined by Qubit fluorometry. The ADP-ribosylation factor gene was used to amplify fragments of 60 and 65 base pairs in length. These were then utilized as size markers to facilitate the visualization and extraction of degradome libraries in the subsequent steps (Figure 1A).

To achieve enhanced separation, high-resolution MetaPhor™ agarose at 4% and 5% concentration was evaluated (Figure 1B,D) [23]. Given the challenges associated with dissolving agarose and preparing vertical gels at a 5% concentration, agarose at a 4% concentration was selected for subsequent testing. To facilitate comparison, sample separation was also performed in a 12% polyacrylamide gel. However, despite the high-resolution images of the products obtained upon visualization, the recovered libraries had very low concentrations (Figure 1C). The 4% high-resolution MetaPhor™ agarose gel was selected as the optimal gel for the separation and visualization of degradome libraries based on the results of the electrophoresis analysis and the final product concentration. The utilization of high-resolution agarose facilitated the observation of size marker bands. Employing high-resolution agarose for separating degradome libraries and using dedicated size markers enabled the observation of a library smear and the precise excision of a gel section containing fragments within the 60–65 bp range. This contrasts with the methodology employed by other research teams, who excised fragments based solely on the size marker [24].

The 60 bp and 65 bp PCR products were cut and retrieved from the gel. DNA fragments were purified using standard laboratory consumables instead of specialized, expensive cellulose acetate or nylon membrane centrifuge tube filters. Therefore, a 1.5 mL Eppendorf tube was used with a 0.2 mL tube placed inside (Supplementary Materials S1 Figure S3). A preparation needle was used to make a hole in the bottom of the 0.2 mL tube. Two layers of sterile autoclavable gauze were placed on the bottom of the smaller tube. A solution containing sodium acetate, ethanol, and glycogen was added to each filtrate to precipitate DNA.

**Figure 2:**
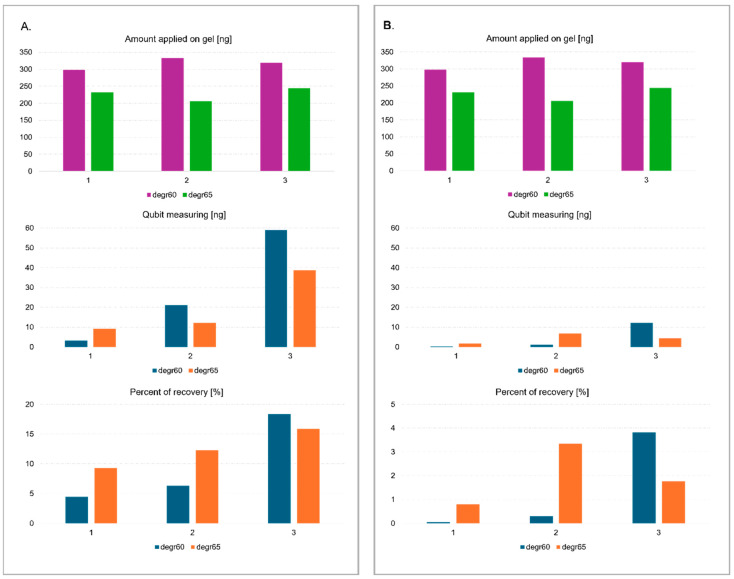
Concentration of 60, 65 bp libraries extracted from: (**A**) high-resolution 4% MetaPhor™ agarose and (**B**) 12% polyacrylamide using one of three purification methods (1. freezing fragments with glycogen added; 2. centrifuging on gauze; 3. centrifuging on gauze with glycogen added).

The solution was enriched by adding glycogen due to the low concentration of fragments in the obtained filtrates. Glycogen is a polysaccharide that can effectively co-precipitate with DNA in alcohol, such as ethanol or isopropanol. It aggregates with DNA, increasing the weight of the molecules and facilitating precipitation [25]. For samples with low DNA concentrations, adding glycogen notably increases the efficiency of the precipitation procedure. Low-concentration DNA can be challenging to precipitate, but glycogen helps form larger, more easily centrifuged aggregates. Thus, even minimal amounts of DNA can be recovered, which might be left in the supernatant without glycogen [26]. Due to their low efficiency for fragments less than 70 bp in length and low initial concentration, none of the commercially available agarose gel elution kits were used. Three methods were tested for eluting and precipitating degradome library fragments after agarose and polyacrylamide gel electrophoresis, i.e., freeze and squeeze method in a glycogen-containing solution, centrifuged in a gauze-containing tube with standard eluent without glycogen, and centrifuged in a gauze-containing tube with a solution enriched with glycogen. The concentration of the DNA fragments was measured before electrophoresis and after elution from the gel using a Qubit fluorimeter and a dsDNA HS Assay Kit (Agilent , Santa Clara, CA, USA). Based on the results, the highest sample recovery of ~20% was obtained using the third method from agarose gel (Figure 2). The efficiency of elution from the polyacrylamide gel was undoubtedly influenced by the type of buffer used. It is standard practice to use high ionic strength buffers containing EDTA in their composition for elution [27,28]. However, the presence of EDTA in samples undergoing next-generation sequencing (NGS) is unfavorable due to the inhibition of MG^2+^ ion-dependent enzymes, such as ligase and polymerase. This can contribute to incomplete or inefficient amplification and ligation, resulting in lower sequencing yield with compromised quality [29,30]. Due to the low concentration of DNA fragments, EDTA could not be removed by further purification steps.

**Figure 3:**
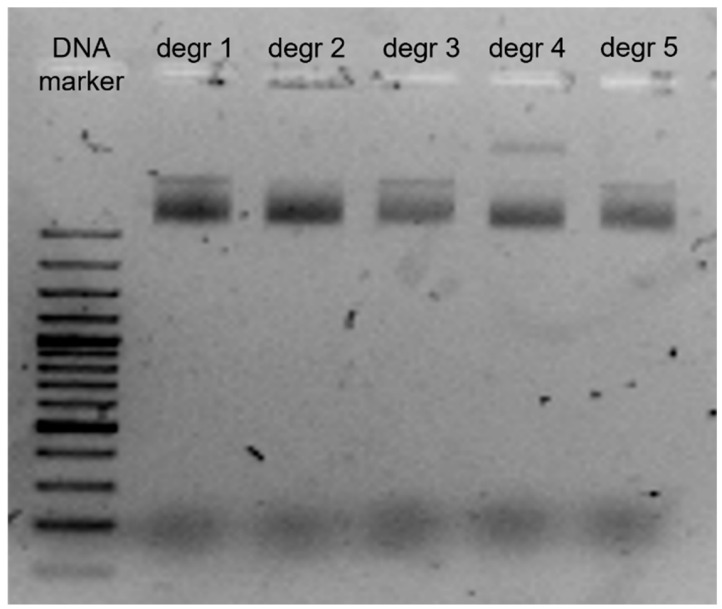
Electrophoretic image of plasmid DNA from *E. coli* (plasmid DNA contained a degradome library insert).

The protocol for generating degradome libraries was validated using genetic engineering tools. The process of validating libraries is described in detail in Supplementary Materials S3. The final fragments of the libraries were cloned into the pGEM-T Easy Vector System vector. The vector was electroporated into competent *Escherichia coli* DH5α bacteria. The pGEM-T Easy Vector System vector uses a plasmid with a length of 3015 bp. The insertion site in the vector is located in the lacZ gene, which encodes the ß-galactosidase subunit. Clones containing the correct insertion were selected by adding X-Gal and ampicillin to the medium. Alkaline lysis was performed to obtain plasmid DNA. For evaluation of the alkaline lysis process, electrophoresis was performed on a 1.5% agarose gel, on which 5 µg of samples were loaded (Figure 3).

**Figure 4:**
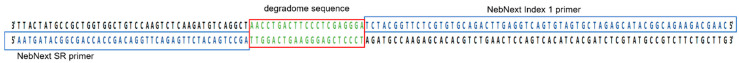
The degradome library sequence was obtained by Sanger sequencing.

Sanger sequencing was performed on the fragments containing the integrated degradome library. The sequences obtained confirmed the correct binding of all adapters to the fragment obtained after restriction with the enzyme *Mme I* (Figure 4).

**Figure 5:**
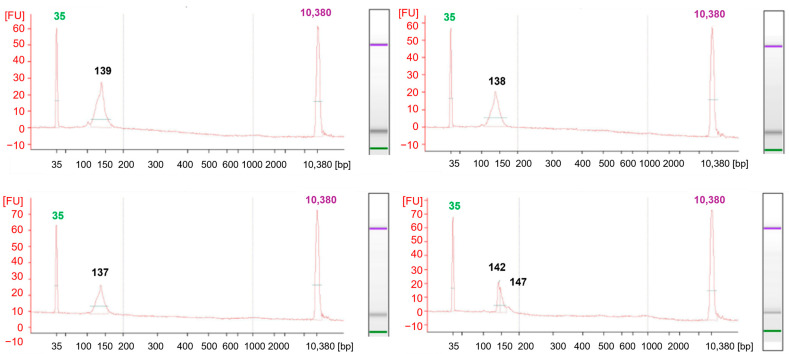
The result of the degradome library construction described in this study was achieved using a 2100 Bioanalyzer High Sensitivity kit (Agilent, Santa Clara, CA, USA). Electrophoregrams describe the library size.

**Figure 6:**
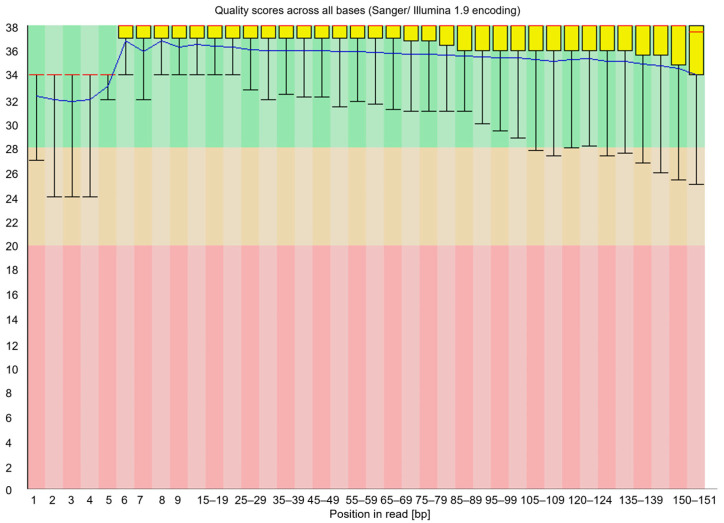
Qualitative analyses of degradome libraries were performed with the FastQC program.

During the analysis, 130–150 bp libraries were confirmed in size using Bioanalyzer 2100 automatic electrophoresis (Figure 5). Using the Mme I enzyme in the method, and a highly effective purification technique involving centrifugation, allows the generation of libraries sequenced as 36 bp. This approach offers a substantial cost reduction compared to conventional protocols. The approach, suggested by Carpentier, for sequencing long fragments of around 150 bp results in higher analysis costs [8]. Shorter reads noticeably reduce the data required for high coverage of the cut sites, which is essential for degradome-seq analyses. In this type of study, key information is concentrated at the 5′ ends of the degradation sites. Long continuous reads are not required [5]. Following the steps described in the protocol, the constructed library produced 25 million single reads using MiSeq Reagent Kit v3 (Illumina, Inc., San Diego, CA, USA). As a result of sequencing, 7791.680, 7858.994, and 6797.471 raw reads were obtained. The high quality of the reads (Figure 6) was confirmed by evaluating the reads obtained during sequencing using FastQC [31]. The parameter indicating the quality of the sequencing (QC) was 98.5%. The qualitative analysis of the raw reads indicated their high quality.

**Figure 7:**
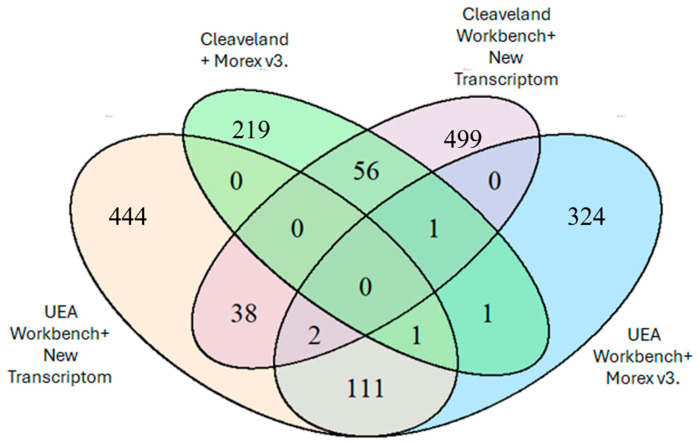
The number of degradome sequences matches the miRNA using various bioinformatics analyses, UEA Workbench, and Cleaveland 4.0 algorithms.

The developed protocol has been used to verify targets for miRNAs detected in dry seeds and during the first 24 h of germination of barley seeds after long-term storage. Further research aims to identify the relationship between germination capacity and miRNAs and seed aging processes. The presented protocol was applied to degradome sequencing analyses of barley seeds during germination, as the sequencing identified potential target genes for miRNAs identified in the study. Figure 7 shows the number of identified degradome sequences mapped to miRNAs, depending on the chosen bioinformatics analysis method. The highest number of identified sequences was obtained using an analysis algorithm based on the CleaveLand 4.0 program and an in-house assembly of the Hordeum vulgare cultivar Damazy transcriptome (596 sequences). Using the UEA Workbench analysis algorithm and the new transcriptome assembly, an identical value of 591 sequences was also obtained.

**Figure 8:**
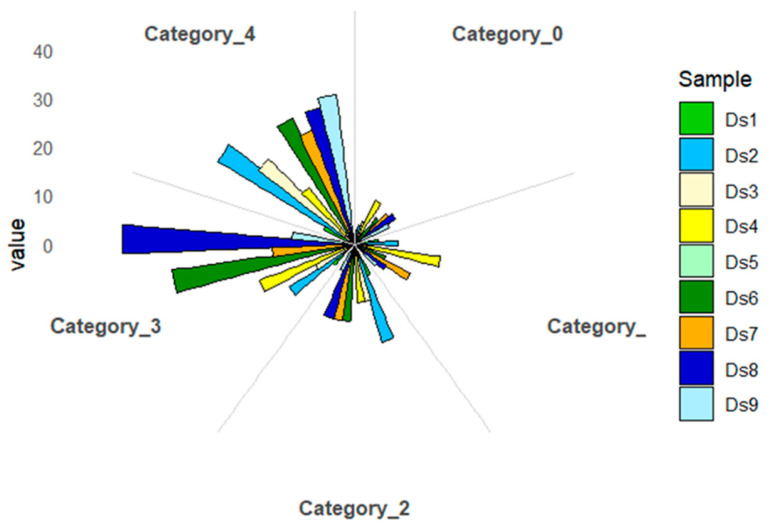
Categorization of degradome sequences using Cleaveland 4.0 software.

The identified degradome sequences were evaluated for class membership based on the number of reads in a given transcript position. (Figure 8). Category 0 includes positions with >1 read, the only maximum in a given transcript, indicating the highest specificity and reliability. Category 1 contains positions with >1 read that co-occur with other positions with the same maximum number of reads, indicating parallel cuts at different mRNA sites. Category 2 includes positions with >1 read that exceed the average number of reads for the transcript, but not the maximum, indicating moderate experimental support. Category 3 is assigned to positions with >1 read equal to or below the average, indicating low confidence, often associated with technical noise or poor target expression. Category 4 includes positions with only one read, which are the least reliable and may represent artifacts. As a result of categorizing the sequences assigned to the cutting sites, more than 25% of the reads matched to category 4 were observed in all the studied samples. About 20% of the sequences in each sample studied were matched to category 3, except sample D_5_. The most significant number of reads matched to category 0 was identified in samples D_4_ and D_9_ (13%) and D_1_ and D_7_ (11%).

The degradome analyses significantly improved the identification of miRNA target sequences in radish, rice, and cotton [32,33]. The preparation of high-quality degradome libraries allowed the verification of false positives from in silico analyses [34]. The developed protocol allowed the construction and sequencing of degradome libraries from challenging material, such as barley grains after long-term storage, which were characterized by uneven degradation of various RNA fractions and low RIN [11]. Moreover, the protocol does not require the purchase of additional reagents. It is based on the ingredients included in the kit for sRNA analysis, thus notably reducing the cost of library construction. Using additional dedicated size markers allows for the precise excision of a band containing the appropriate length of fragments from the gel. Compared to previously used methods based on polyacrylamide gels, optimizing the purification method of degradome-seq libraries increased the yield of fragments obtained [9]. The protocol also saves considerable time by completing the library preparation within two days. The degradome library construction method proposed by German et al. (2009) requires material with a concentration of 5–20 µg and RIN > 7 and 6 days, while Li et al. (2017) proposed a method based on 200 μg of RNA. The methodology developed in the study allows analysis on degraded material with RIN < 3 and a concentration of about 1 μg RNA [5,35].

This study delineates an optimized and cost-efficient degradome sequencing (degradome-seq) protocol that facilitates the construction of high-quality libraries from highly degraded RNA samples exhibiting RNA Integrity Number (RIN) values below 3, utilizing as little as 1 µg of total RNA. The protocol incorporates several methodological innovations, notably the reutilization of residual reagents from small RNA (sRNA) sequencing library kits, thereby substantially reducing associated costs. Additionally, it introduces a novel purification strategy employing 4% MetaPhor™ high-resolution agarose gel coupled with gauze-based centrifugation, which enables the efficient recovery of short fragments (60–65 base pairs) with an average yield of approximately 20%. Crucially, this method obviates the need for duplex-specific nuclease (DSN)-based ribosomal RNA depletion by relying on poly(A)+ mRNA selection, simplifying the workflow and further decreasing expenses. The quality of the constructed libraries was validated through Bioanalyzer profiling, Qubit fluorometry, and Sanger sequencing. Subsequent Illumina sequencing yielded over 25 million reads, even from long-term stored barley seed samples characterized by poor RNA integrity. Given its minimal reagent requirements, brief processing time (two days), and compatibility with low-quality RNA, this protocol is particularly advantageous for laboratories with limited financial resources, especially those possessing residual reagents from sRNA library preparations. Consequently, it provides an accessible, robust, and efficient tool for degradome profiling applicable across a broad spectrum of plant research contexts.

## 3. Materials and Methods

**Figure 9:**
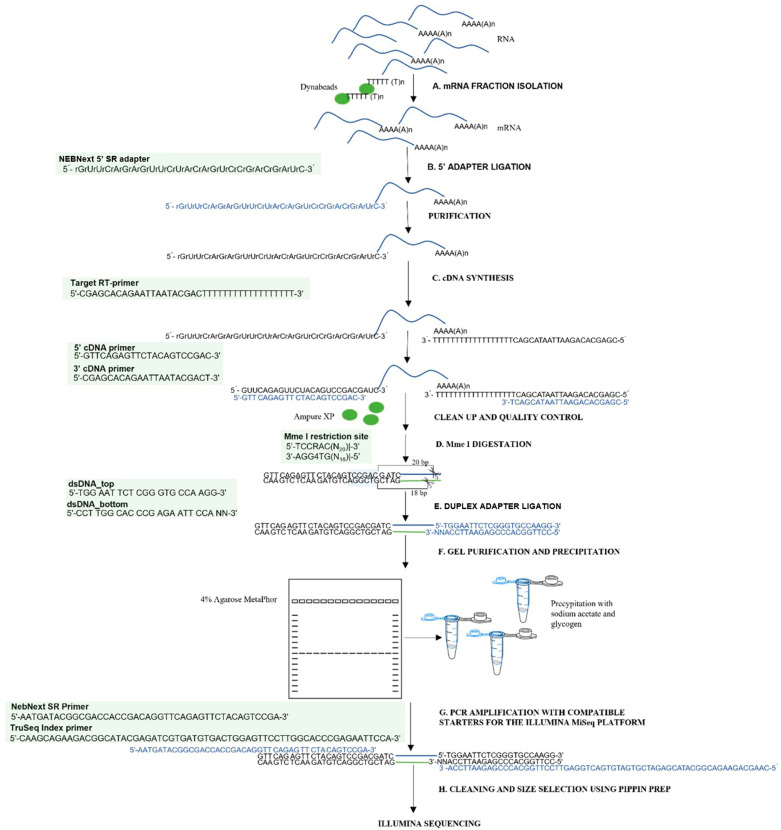
The scheme for constructing a degradome library. The procedure comprises the following steps: (**A**) mRNA fraction isolation, (**B**) 5’ adapter ligation, (**C**) cDNA synthesis, (**D**) Mme I digestion, (**E**) duplex adapter ligation, (**F**) gel purification and precipitation, (**G**) PCR amplification with Illumina primers, and (**H**) cleaning and size selection library using Pippin Prep.

To enable the efficient analysis of the RNA degradome in low-quality samples, a modified degradome-seq library preparation protocol was developed and implemented. This protocol is based on optimizing existing procedures and reusing reagents from the small RNA analysis kit. The procedure involves steps from isolating mRNA fractions to acquiring finished libraries suitable for sequencing on the Illumina platform. To clarify the method and allow it to be replicated, Figure 9 shows a complete flowchart of degradome library preparation, visualizing the subsequent steps of the protocol, including adapter ligation, cDNA synthesis, enzymatic restriction, fragment purification, and final amplification. The whole protocol is described in detail in Supplementary Materials S1. All necessary reagents and equipment are listed in Supplementary Materials S2.

### 3.1. Plant Material and RNA Extraction

Barley *(Hordeum vulgare* L.) grains were the biological source for total RNA extraction. The material was derived from seeds subjected to long-term storage, and all analyses were conducted in three independent biological replicates. Puchta et al. described the plant material in detail [21]. For RNA isolation, the embryonic axis, encompassing the embryo and scutellum, was carefully dissected from dry seeds. Approximately 100 mg of finely ground tissue was processed per replicate. Total RNA was extracted utilizing TRIzol^®^ reagent (Thermo Fisher Scientific, Oxford, UK) according to a modified version of the manufacturer’s protocol, optimized explicitly for plant tissues characterized by high levels of storage compounds. RNA purity and concentration were quantified using a Qubit™ fluorometer with the RNA HS Assay Kit (Agilent , Santa Clara, CA, USA), whereas RNA integrity was assessed via the Agilent 2100 Bioanalyzer employing either the RNA 6000 Nano or Pico Kit (Agilent, Santa Clara, CA, USA). Comprehensive details regarding reagent volumes and centrifugation conditions are provided in Supplementary Materials S1.

### 3.2. mRNA Enrichment and Library Construction

Polyadenylated [poly(A)+] mRNA was isolated from total RNA using oligo(dT)-conjugated magnetic beads with minor procedural modifications implemented to improve yield from degraded samples exhibiting RNA integrity numbers (RIN) below 3. Degradome libraries were constructed following a protocol incorporating reagent recycling and an optimized purification strategy. Residual reagents from the NEBNext^®^ Small RNA Library Prep Kit (New England Biolabs, Ipswich, MA, USA) were reutilized, substantially reducing the overall cost of library preparation. The library preparation workflow included the following steps: ligation of a 5′ adapter to the poly(A)+ RNA, reverse transcription using a poly(T) primer, digestion with the restriction enzyme Mme I to generate uniform 3′ ends, ligation of a double-stranded 3′ adapter, PCR amplification employing primers compatible with Illumina sequencing platforms, and precise size selection of the final libraries. Detailed information regarding buffer compositions, reaction volumes, and incubation parameters is provided in Supplementary Materials S1.

### 3.3. Gel Purification and Library Quality Control

PCR-amplified libraries were purified using 4% MetaPhor™ high-resolution agarose gel (Lonza, Basel, Switzerland). Custom-size markers of 60 and 65 bp, generated by amplifying an ADP-ribosylation factor fragment (GenBank accession number 838957), facilitated precise excision of the target DNA smear. DNA fragments were subsequently recovered by centrifugation through gauze-lined tubes, followed by ethanol precipitation in the presence of sodium acetate and glycogen. For comparative evaluation, alternative separation and purification methods were also assessed, including electrophoresis in 2.5 agarose gel, 5% MetaPhor™ high-resolution agarose gel, and 12% polyacrylamide gel, as well as freezing gel fragments in the presence of glycogen and centrifuging on gauze without glycogen. The quality and size distribution of the final libraries were confirmed using an Agilent 2100 Bioanalyzer, and library concentrations were determined by Qubit fluorometry.

### 3.4. Illumina Sequencing

Final libraries were denatured, diluted to a concentration of 20 pM, and spiked with 1% PhiX control. Sequencing was conducted on the Illumina (San Diego, CA, USA) MiSeq platform utilizing the MiSeq Reagent Kit v3 (50 cycles) (Illumina, Inc., San Diego, CA, USA), with a single-end read length of 36 bp. Run setup, cluster generation, and data acquisition were conducted, following the manufacturer’s protocols.

### 3.5. Bioinformatic Analysis

Raw sequencing reads underwent quality assessment using FastQC 0.12.0 software (v0.11.9). Adapter sequences were trimmed, and low-quality reads were filtered using Cutadapt 5.1. Degradome tags were aligned to the Hordeum vulgare cv. Damazy reference transcriptome employing two independent pipelines: CleaveLand 4.0 and the UEA sRNA Workbench 4.4. Cleavage sites were classified according to the established five-category system (categories 0–4), based on read abundance and positional distribution within target transcripts.

## Data Availability

No new data were created or analyzed in this study.

## Supplementary Materials

The following supporting information can be downloaded at: https://www.mdpi.com/article/10.3390/ijms26147020/s1.

## References

[1] Hackenberg M., Gustafson P., Langridge P., Shi B.J. (2015). Differential expression of microRNAs and other small RNAs in barley between water and drought conditions. Plant Biotechnol. J..

[2] Rhoades M.W., Reinhart B.J., Lim L.P., Burge C.B., Bartel B., Bartel D.P. (2002). Prediction of plant microRNA targets. Cell.

[3] Qiao Y., Yang F., Li Q., Ren P., An P., Li D., Xiao J. (2023). Combined Small RNA and Degradome Sequencing Reveals Important Roles of Light-Responsive microRNAs in Wild Potato (*Solanum chacoense*). Agronomy.

[4] Zhai J., Arikit S., Simon S.A., Kingham B.F., Meyers B.C. (2014). Rapid construction of parallel analysis of RNA end (PARE) libraries for Illumina sequencing. Methods.

[5] German M.A., Luo S., Schroth G., Meyers B.C., Green P.J. (2009). Construction of Parallel Analysis of RNA Ends (PARE) libraries for the study of cleaved miRNA targets and the RNA degradome. Nat. Protoc..

[6] Addo-Quaye C., Eshoo T.W., Bartel D.P., Axtell M.J. (2008). Endogenous siRNA and miRNA targets identified by sequencing of the *Arabidopsis* degradome. Curr. Biol..

[7] Yu X., Willmann M.R., Anderson S.J., Gregory B.D. (2016). Genome-wide mapping of uncapped and cleaved transcripts reveals a role for the nuclear mRNA cap-binding complex in cotranslational RNA decay in Arabidopsis. Plant Cell.

[8] Carpentier M.C., Bousquet-Antonelli C., Merret R. (2021). Fast and Efficient 5’P Degradome Library Preparation for Analysis of Co-Translational Decay in Arabidopsis. Plants.

[9] Lin S.S., Chen Y., Lu M.J. (2019). Degradome Sequencing in Plants. Methods Mol. Biol..

[10] Guo L., Li Y., Zhang C., Wang Z., Carlson J.E., Yin W., Zhang X., Hou X. (2022). Integrated analysis of miRNAome transcriptome and degradome reveals miRNA-target modules governing floral florescence development and senescence across early- and late-flowering genotypes in tree peony. Front. Plant Sci..

[11] Puchta M., Boczkowska M., Groszyk J. (2020). Low RIN value for RNA-seq library construction from long-term stored seeds: A case study of barley seeds. Genes.

[12] Yu Y., Jia T., Chen X. (2017). The ‘how’ and ‘where’ of plant microRNAs. New Phytol..

[13] German M.A., Pillay M., Jeong D.H., Hetawal A., Luo S., Janardhanan P., Kannan V., Rymarquis L.A., Nobuta K., German R. (2008). Global identification of microRNA-target RNA pairs by parallel analysis of RNA ends. Nat. Biotechnol..

[14] Petrov A., Wu T., Puglisi E.V., Puglisi J.D. (2013). RNA purification by preparative polyacrylamide gel electrophoresis. Methods Enzymol..

[15] Ma X., Yin X., Tang Z., Ito H., Shao C., Meng Y., Xie T. (2020). The RNA degradome: A precious resource for deciphering RNA processing and regulation codes in plants. RNA Biol..

[16] Wang Y., Hu Q., Yao Y., Cui Y., Bai Y., An L., Li X., Ding B., Yao X., Wu K. (2025). Transcriptome, miRNA, and degradome sequencing reveal the leaf stripe (*Pyrenophora graminea*) resistance genes in Tibetan hulless barley. BMC Plant Biol..

[17] Sega P., Kruszka K., Bielewicz D., Karlowski W., Nuc P., Szweykowska-Kulinska Z., Pacak A. (2021). Pi-starvation induced transcriptional changes in barley revealed by a comprehensive RNA-Seq and degradome analyses. BMC Genom..

[18] Yue H., Zhang H., Su N., Sun X., Zhao Q., Weining S., Nie X., Yue W. (2022). Integrate Small RNA and Degradome Sequencing to Reveal Drought Memory Response in Wheat (*Triticum aestivum* L.). Int. J. Mol. Sci..

[19] Chen A.P., Chen W.C., Hou B.H., Chou S.J., Chen H.M. (2025). Global Profiling and Analysis of 5’ Monophosphorylated mRNA Decay Intermediates. Methods Mol. Biol..

[20] Yu D., Wan Y., Ito H., Ma X., Xie T., Wang T., Shao C., Meng Y. (2019). PmiRDiscVali: An integrated pipeline for plant microRNA discovery and validation. BMC Genom..

[21] Puchta M., Groszyk J., Małecka M., Koter M.D., Niedzielski M., Rakoczy-Trojanowska M., Boczkowska M. (2021). Barley Seeds miRNome Stability during Long-Term Storage and Aging. Int. J. Mol. Sci..

[22] Zhang Y., Pelechano V. (2021). High-throughput 5’P sequencing enables the study of degradation-associated ribosome stalls. Cell Rep. Methods.

[23] Ordovas J.M. (1998). Separation of small-size DNA fragments using agarose gel electrophoresis. Methods Mol. Biol..

[24] Hou C.Y., Wu M.T., Lu S.H., Hsing Y.I., Chen H.M. (2014). Beyond cleaved small RNA targets: Unraveling the complexity of plant RNA degradome data. BMC Genom..

[25] Tan S.C., Yiap B.C. (2009). DNA, RNA, and protein extraction: The past and the present. BioMed Res. Int..

[26] Ausubel F.M., Brent R., Kingston R.E., Moore D.D., Seidman J. (1992). Short Protocols in Molecular Biology.

[27] Maniatis T. (1982). Molecular Cloning: A Laboratory Manual.

[28] Maxam A.M., Gilbert W. (1977). A new method for sequencing DNA. Proc. Natl. Acad. Sci. USA.

[29] Illumina (2018). Nextera XT DNA Library Prep Kit Reference Guide.

[30] Lecker D.N., Khan A. (1996). Theoretical and Experimental Studies of the Effects of Heat, EDTA, and Enzyme Concentration on the Inactivation Rate of α-Amylase from Bacillus sp. Biotechnol. Prog..

[31] Leggett R.M., Ramirez-Gonzalez R.H., Clavijo B.J., Waite D., Davey R.P. (2013). Sequencing quality assessment tools to enable data-driven informatics for high throughput genomics. Front. Genet..

[32] Hu J., Jin J., Qian Q., Huang K., Ding Y. (2016). Small RNA and degradome profiling reveals miRNA regulation in the seed germination of ancient eudicot Nelumbo nucifera. BMC Genom..

[33] Yan X., Li C., Liu K., Zhang T., Xu Q., Li X., Zhu J., Wang Z., Yusuf A., Cao S. (2024). Parallel degradome-seq and DMS-MaPseq substantially revise the miRNA biogenesis atlas in Arabidopsis. Nat. Plants.

[34] He D., Wang Q., Wang K., Yang P. (2015). Genome-Wide Dissection of the MicroRNA Expression Profile in Rice Embryo during Early Stages of Seed Germination. PLoS ONE.

[35] Li R., Chen D., Wang T., Wan Y., Li R., Fang R., Wang Y., Hu F., Zhou H., Li L. (2017). High throughput deep degradome sequencing reveals microRNAs and their targets in response to drought stress in mulberry (*Morus alba*). PLoS ONE.

