# Supplementary material for: Improved Degradome Sequencing Protocol via Reagent Recycling from sRNAseq Library Preparations
Source: Int J Mol Sci. 2025 Jul 21;26(14):7020. doi: 10.3390/ijms26147020 (PMC12295840; doi:10.3390/ijms26147020)
Supplement: Supplementary file 1 [file ijms-26-07020-s001.zip › Supplementary File S1.pdf]

Comparison of RNA Degradome Library Preparation Methods

Feature / Method	PARE [5]	GMUCT [7]	5P-seq [22]	Degradome-seq [9]	New protocol
Minimum RNA quality (RIN)	≥7.0	≥ 7.0	Not specified	≥7.0 recommended; ≥8.0 preferred	Tolerates severely degraded RNA, RIN < 3
Minimum RNA input	20–30 µg total RNA; ~5 µg mRNA	20–50 µg total RNA	6 µg total RNA	80–100 µg total RNA	1–5 µg total RNA from degraded plant tissue
Library preparation time	6–7 days	2–3 days	1-2 days	~4 days	≤2 days;
Estimated cost	High – multiple specialized reagents	Medium–High	Medium - RNA depetion, Duplex-specific nuclease	High – dedicated Illumina kits	Low – reuses leftover reagents from sRNA-seq kits
Fragment purification step	PAGE + 0.45-µM filter	PAGE + 0.45-µM filter	AMPure beads	PAGE + 0.45-µM filter	MetaPhor™ agarose + gauze-filtration
Fragment recovery yield [ng}	Not specified	Not specified	Not specified	0.8-7	59
Required fragment length	~20–21 nt insert (MmeI); final product ~86 bp	20–200 nt	20-50 nt insert; ~120nt final	~20–21 nt insert (MmeI); final product 60–65 bp	~20–21 nt insert (MmeI); precisely 60–65 bp; size defined using custom markers
Adaptable to degraded RNA?	No	Partially adaptable	Yes	No	Yes – explicitly developed for low-quality RNA
rRNA depletion required?	No – poly(A)+ selection	Yes	Yes	No – poly(A)+ selection	No – poly(A)+ selection
Sequencing platform	Illumina GAII, HiSeq	Illumina HiSeq/MiSeq	Illumina NextSeq, HiSeq	Illumina MiSeq, NextSeq, HiSeq	Illumina MiSeq/HiSeq, 36 bp single-end reads
Use of size markers	Yes - 10 bp DNA ladder	Yes - 25 bp DNA ladder	No	Yes – 10/25 bp DNA ladders	Yes – 60/65 bp markers from ADP-ribosylation factor
