# Supplementary material for: Improved Degradome Sequencing Protocol via Reagent Recycling from sRNAseq Library Preparations
Source: Int J Mol Sci. 2025 Jul 21;26(14):7020. doi: 10.3390/ijms26147020 (PMC12295840; doi:10.3390/ijms26147020)
Supplement: Supplementary file 1 [file ijms-26-07020-s001.zip › Supplementary File S2.pdf]

Libraries validation protocol

Library cloning was performed using Escherichia coli DH5α competent bacteria and the pGEM-T Easy Vector System vector. In the vector, the insertion site is located in the lacZ gene, encoding the ß-galactosidase subunit. Selection of clones with the correct insertion is performed by adding X-Gal and ampicillin to the substrate.

1. A-tilling (addition of adenine at the 3' end) was performed first, for which a reaction was prepared with the following composition (Table 1)

2. Total volume (3 µl), was incubated at 70 °C for 30 minutes.

Table S1. Composition of the A-tilling reaction

Reagent	Volume
library degradome-Seq	3,2 ng
2mM dATP	0,2 mM
10x DreamTaq buffer	1x
5U/µl DNA DreamTaq polimerase	1,5 U
total	3 µl

3. Prepared the ligation of the degradome libraries to the plasmid mix, shown in Table 2.

Table S2. Composition of the ligation library to plasmid reaction

Reagent	Volume
A-tiling reaction	30%
pGEM T-easy plasmide	10%
T4 ligase	3U
2x buffer	1x
total	10 µl

4. Reaction incubated at 16 °C overnight.
5. Plasmids obtained by electroporation were transformed into competent cells of E.coli, strain DH5α (electrical pulses of 2.5 kV).
6. Bacteria were incubated in SOC medium, pH 7 (Table 3) in a thermoblock at 37 °C for 1 h at 250 rpm.

Table S3. Composition SOC medium

Reagent	Concentration
tryptone	2%
yeast extract	0,5%
NaCl	8,6 mm
$MgCl_2$	10 mM
glucose	20 mM
KCl	2,5 mM
bacteriological agar	1%

7. 200 µl of bacteria were inoculated onto LB solid medium (Table 4) with ampicillin, pH 7,
8. Addition of selection factors 0.1mM X-Gal and 6.9 µM IPTG, and incubated at 37 °C overnight.

Table S4. Composition LB medium with ampiciline and X-Gal, IPTG

Reagent	Concentration
tryptone	1%
yeast extract	0,5%
NaCl	172 mM
KCl	13 mM
Bacteriological agar	1%
ampicyline	286 mM

9. Reduction cultures were made from white bacterial colonies on LB media with ampicillin and incubated at 37 °C overnight.
10. Selected bacterial colonies were transferred to 3 ml of LB liquid media with ampicillin (Table 5).
11. Bacterial culture was conducted at 37 °C overnight at 250 rpm.

Table S5. Composition LB medium

Reagent	Concentration
tryptone	1%
yeast extract	0,5%
NaCl	172 mM
KCl	13 mM
ampicyline	286 mM

12. The bacterial culture was cooled on ice and centrifuged at 22 °C for 1 min at 13 000 rpm.
13. Supernatant was removed, the pellet was resuspended in GTE buffer, pH 8 (Table 6),
14. Next 5 µl RNase was added, mixed gently and incubated at 22 °C for 5 min.

Table S6. Composition GTE buffer

Reagent	Concentration
glucose	50 mM
Tris HCl	25 mM
EDTA	10 mM

15. 200 µl of a 1% solution of SDS with 0.2 M NaOH was added. Gently mixed by rotating the tubes and placed on ice.
16. After 5 minutes of incubation, 150 µl of cold solution III with the composition shown in Table 7 was added to the homogeneous, transparent lysate.

Table S7. Composition III lise buffer

Reagent	Concentration
5 M KoAc	0,3 mM
cold acetic acid 99,5%	11,5 mM

17. The mixture was then shaken vigorously and left on ice for 10 minutes.
18. The mixture was centrifuged at 22 °C for 10 minutes at 13 000 rpm.
19. The supernatant was removed and the pellet was washed with 500 µl of 70% ethanol.
20. The pellet was centrifuged at 22 °C for 10 minutes at 13,000 rpm, dried and dissolved in 30 µl of DNAz and RNAz free water for one hour in the refrigerator.
21. Next PCR reaction was then performed with two pairs of primers, T7 and SP6, and M13 (Table 8) using the HGC PCR Mix Plus kit with the following composition (Table 9) and thermal profile (Figure 1).

Table S8. Primer sequences

Primer	Sequence
T7	5’ TAATACGACTCACTATAGGG 3’

SP6	5′ ATTTAGGTGACACTATAGAA 3′
M13_R	5′ GTAAAACGACGGCCAGT 3′
M13_F	5′ CAGGAAACAGCTATGAC 3′

Table S9. Composition PCR Mix Plus HGC

Reagent	Concentration
PCR Mix Plus HGC 2x	1x
forward primer	0,4 μM
revers primer	0,4 μM
DNA template	50 ng
H_2O	to final volume
Final volume	10 μl

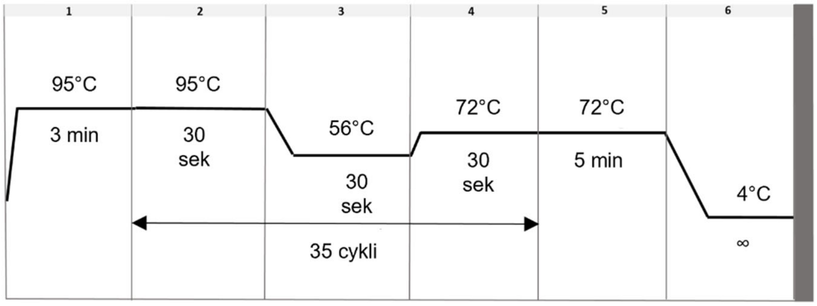

Figure S1. Thermal profile PCR HGC reaction

22. Reaction products were separated electrophoretically (1.5% agarose gel and visualised in the presence of Midori Green dye under UV light (λ=312 nm) using a Proxima C16 imaging system).
23. PCR products were purified using Eppic Fast.
24. Sample concentrations were measured using a Qubit fluorimeter (Qubit dsDNA HS Assay Kit)
25. Sample diluted to 5 ng/μl (according to the manufacturer's protocol for sequencing reactions of 100-200 nt fragments using the BrightDye Terminator Cycle Sequencing Kit).
26. Sequencing PCR was performed according to Table 16 and the thermal profile according to Figure 2.

Table S10. Composition Sanger sequencing with BrightDye Terminator Cycle Sequencing Kit (MCLAB)

Reagent	Volume
Bright Dye	5%
5x sequencing Buffer	0,875x
primer	3,2 pM
DNA template	5 ng
H_2O	to final volume
final volume	10 μl

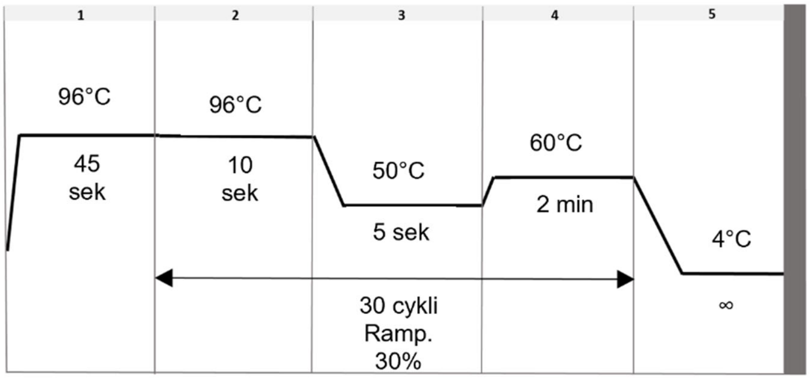

Figure S2. Thermal profile Sanger sequencing PCR

27. PCR products were purified with the ExTerminator kit.
28. Purified products were denatured at 80 °C for 3 min and then sequenced on a 3130 XL Genetic Analyzer capillary sequencer using 36 cm capillaries and NanoPOP-7 polymer.
