# Supplementary material for: Improved Degradome Sequencing Protocol via Reagent Recycling from sRNAseq Library Preparations
Source: Int J Mol Sci. 2025 Jul 21;26(14):7020. doi: 10.3390/ijms26147020 (PMC12295840; doi:10.3390/ijms26147020)
Supplement: Supplementary file 1 [file ijms-26-07020-s001.zip › Supplementary File S3.pdf]

List of reagents and equipment

1. Reagents

Reagent	Reagent Manufacturer	Catalog number
TRI Reagent	Sigma-Aldrich, Saint Louis, MO, USA	T9424
1-bromo-3-chloropropane	Sigma-Aldrich, Saint Louis, MO, USA	B9673
2-propanol	POCH, Gliwice, Poland	751503420
99,8% ethanol A.C.S.	Chempur, Piekary Śląskie, Poland	113964800
Molecular biology water non-treated with DEPC	A&A Biotechnology, Gdynia, Poland	003-075
KoAc	Sigma-Aldrich, Saint Louis, MO, USA	P1190
Dynabeads Purification Kit for mRNA	Invitrogen, Carlsbad, CA, USA	61006
NebNext small RNA Library Prep Set for Illumina	New England Biolabs, Ipswich, MA, USA	E7300S
Ribolock (40 U/μL)	ThermoFisher Scientific, Waltham, MA, USA	E00381
Maxima H Minus First Strand cDNA Synthesis Kit (200 U)	ThermoFisher Scientific, Waltham, MA, USA	K1652
AmPure XP for PCR Purification	Beckman Coulter Life Sciences; Indianapolis, United States	A63880
SeqAmp DNA Polymerase	Takara Bio, Otsu, Japan;	638504
Customer primers (10 μM)		
Phix Control Sequencing v3	Illumina, San Diego, CA, USA	FC-110-3001
Illumina MiSeq, Reagent Kit v3 (50-cycles) cartridge	Illumina, San Diego, CA, USA	MS-102-2001
NaOH	PolAura, Morąg, Poland	1310-73-2
Sodium acetate (Sigma-Aldrich, Saint Louis, MO, USA; P1190)		P1190
Glycogen	ThermoFisher Scientific, Waltham, MA, USA	R0561
Tris (Tris(hydroxymethyl-aminomethane))	ROTH CARL, Karslsruhe, Germany	48255.2
EDTA	WARCHEM, Warsaw, Poland	0566.06
Acetic acid A.C.S. 80%	StanLab, Lublin, Poland	04/56419730
Qubit dsDNA HS Assay Kit	ThermoFisher Scientific, Waltham, MA, USA	Q33230
Midori Green DNA	Nippon Genetics, Duren, Niemcy	
DNA Gel Loading Dye 6x	ThermoFisher Scientific, Waltham, MA, USA	R0611
T4 DNA Ligase (5U/μl)	Promega, Madison, USA	M180A
Metaphor agarose	Lonza, Basel, Switzerland	733-1211

Mme I (2U/µl)	New England Biolabs, Ipswich, MA, USA	R0637S
Agilent RNA 6000 NanoKit	Agilent, Santa Clara, CA, USA	5067-1511
Agilent High Sensitivity DNA Kit	Agilent, Santa Clara, CA, USA	5067-4626
3% Marker C cartridges Pippin Prep	SageScience, Beverly, MA, USA	CSD3010
DNA ladder 100 bp	ThermoFisher Scientific, Waltham, MA, USA	SM0323
DNA ladder low range	ThermoFisher Scientific, Waltham, MA, USA	SM1191

2. Equipment

Equipment	Equipment Manufacturer	Catalog number
Porcelain mortar	Bionovo, Legnica, Poland	S-1522
Centrifuge	Sigma, Osterode, Germany	Sigma 3-16L
MagJET Separation Rack	ThermoFisher Scientific, Waltham, MA, USA	MR02
ThermoShaker thermoblock TS-100C	Biosan, Riga, Lotwa	010143-1301-0021
Deep freezer -86°C	Phcbi, Breda, Nederlands	MDF-1156-PE
Thermocycler	Applied biosystem, Waltham, MA, USA	4375305
Illumina MiSeq	Illumina, San Diego, CA, USA	SY-410-1003
Qubit 3 Fluorymetr	Invitrogen, Carlsbad, CA, USA	Q33216
Bioanalyzer 2100	Agilent Technologies, Santa Clara, USA	G2939BA
Pippin Prep	SageScience, Beverly, MA, USA	DE54107946
Electrophoresis system vertical gel	Bio-Rad Laboratories, Hercules, USA	1656019

3. Adapter and Customer primer sequence.

Primers	Sequence
target RT-primer	5'-CGA GCA CAG AAT TAA TAC GAC TTT TTT TTT TTT TTT TTT-3'
5' cDNA PCR primer	5'-GTT CAG AGT TCT ACA GTC CGA C-3'
3' cDNA PCR primer	5'-CGA GCA CAG AAT TAA TAC GAC T-3'
dsDNA_top	5'-TGG AAT TCT CGG GTG CCA AGG-3'
dsDNA_bottom	5'-CCT TGG CAC CCG AGA ATT CCA NN-3'
NebNext SR Primer	5'-AAT GAT ACG GCG ACC ACC GAC AGG TTC AGA GTT CTA CAG TCC GA-3'
