# Supplementary material for: Improved Degradome Sequencing Protocol via Reagent Recycling from sRNAseq Library Preparations
Source: Int J Mol Sci. 2025 Jul 21;26(14):7020. doi: 10.3390/ijms26147020 (PMC12295840; doi:10.3390/ijms26147020)
Supplement: Supplementary file 1 [file ijms-26-07020-s001.zip › Supplementary File S4.pdf]

Detailed protocol for degradome sequencing via reagent recycling from sRNAseq Library preparation

Total RNA Isolation

- ❑ Dissect the embryo and scutellum from dry barley seeds (25 seeds per biological replicate).
- ❑ Homogenize the tissue in liquid nitrogen using a pre-cooled porcelain mortar and pestle until a fine powder is obtained.
- ❑ Transfer approximately 100 mg of the powdered tissue into a pre-chilled 2 mL RNase-free microcentrifuge tube.
- ❑ Add 1.0 mL of TRIzol® reagent to the tube, vortex briefly, and incubate at room temperature (23 °C) for 5 minutes.
- ❑ Add 0.1 mL of 1-bromo-3-chloropropane to the mixture, mix vigorously by inversion for 15 seconds, and incubate for 5 minutes at room temperature.
- ❑ Centrifuge the samples at 17,700 × *g* for 10 minutes at 4 °C.
- ❑ Carefully transfer the upper aqueous phase (~500–600 µL) to a new RNase-free microcentrifuge tube, add 0.5 mL of isopropanol, mix gently, and incubate for 10 minutes at room temperature.
- ❑ Centrifuge at 17,700 × *g* for 10 minutes at 4 °C to pellet the RNA.
- ❑ Discard the supernatant and wash the RNA pellet with 1.0 mL of 75% ethanol. Centrifuge at 6,900 × *g* for 5 minutes at 4 °C.
- ❑ Remove the ethanol completely, air-dry the pellet at room temperature (5–10 minutes), and resuspend it in 100 µL of RNase-free molecular biology-grade water.
- ❑ Determine RNA concentration and integrity using a Qubit™ fluorometer (RNA HS Assay Kit) and Agilent 2100 Bioanalyzer (RNA 6000 Nano/Pico Kit), following the manufacturers' protocols.

Poly(A)+ mRNA Enrichment Using Dynabeads™

- ❑ Vortex beads suspension thoroughly and transfer 200 µL to a 2 mL RNase-free microcentrifuge tube.
- ❑ Place the tube on a magnetic stand for 30 seconds. Once the beads are collected against the side of the tube, carefully remove and discard the supernatant.
- ❑ Add 100 µL Binding Buffer to the tube to equilibrate the magnetic beads. Place the tube back onto the magnetic stand and allow the beads to separate. Carefully remove and discard the supernatant.
- ❑ Add 100 µL Binding Buffer followed by 100 µL total RNA (~80 µg), and mix gently by pipetting up and down. Incubate the mixture at room temperature for 5 minutes to allow hybridization.
- ❑ Place the tube back on the magnetic stand until the solution becomes completely clear and the beads are fully collected. Carefully remove and discard the supernatant.
- ❑ Wash the beads twice by adding 200 µL Wash Buffer, mixing gently, and placing the tube on the magnetic stand. After each wash, discard the supernatant without disturbing the pellet.
- ❑ After the final wash, keep the tube on the magnetic stand and allow the beads to air-dry for approximately 1 minute.
- ❑ To elute the captured poly(A)+ RNA, add 20 µL of RNase-free molecular biology-grade water to the dried beads. Mix gently by pipetting and incubate on a thermoblock at 65 °C for 2 minutes with gentle shaking (31 × *g*).
- ❑ Immediately place the tube back on the magnetic stand. Once the beads are fully separated and the solution is clear, transfer 20 µL of the supernatant containing eluted poly(A)+ RNA to a pre-chilled RNase-free microcentrifuge tube (pre-cooled on ice).

5′ adapter ligation

- ❑ Resuspend the lyophilized 5′SR adapter in 120 µL of RNase-free molecular biology-grade water. Mix thoroughly and aliquot as needed.
- ❑ Store the resuspended adapter at –80 °C until use.
- ❑ Immediately prior to ligation, denature the required volume of 5′SR adapter by incubating at 70 °C for 2 minutes. After incubation, place the tube on ice and use the adapter within 30 minutes. Do not vortex or mix after denaturation.
- ❑ Prepare the 5′ adapter ligation reaction as described in Table S1. Due to the foaming tendency of the enzyme mixture, prepare 10% more reaction volume than required to ensure sufficient recovery of the complete reaction mix.
- ❑ Incubate the ligation reaction at 37 °C for 50 minutes.
- ❑ Following ligation, add 80 µL of RNase-free water to the reaction mixture and incubate at 65 °C for 10 minutes to inactivate the enzymes.
- ❑ Immediately place the tube on ice to halt the reaction. Proceed directly to the purification step.

Table S1. Composition of the 5' adaptor ligation reaction

**Reagent**	**Volume (µl)**	**Final concentration**
poly(A)RNA	15 µl	0.3 µg
5′SR adaptor (200µM)	1 µl	10 µM
5′Ligation Reaction Buffer (10x)	1 µl	0.5x
5′Ligation Enzyme Mix	2.5 µl	12.5%
Ribolock (40 U/µL)	0.25 µl	10U
**Total**	**20µl**	**-**

Purification of 5′SR–poly(A)+ RNA Using Magnetic Beads

- ❑ Vortex beads suspension (Dynabeads™ mRNA Purification Kit, Invitrogen) thoroughly and transfer 200 µL to a 2 mL RNase-free microcentrifuge tube.
- ❑ Place the tube on a magnetic stand for 30 seconds. Once the beads are collected against the side of the tube, carefully remove and discard the supernatant.
- ❑ Add 100 µL Binding Buffer to the tube to equilibrate the magnetic beads. Place the tube back onto the magnetic stand and allow the beads to separate. Carefully remove and discard the supernatant.
- ❑ Add 100 µL of Binding Buffer to the beads, followed by 100 µL of 5′SR–poly(A)+ RNA (from the ligation step). Mix gently by pipetting up and down to resuspend the beads completely.
- ❑ Incubate the mixture at room temperature for 5 minutes to allow hybridization.
- ❑ Place the tube back on the magnetic stand until the solution becomes completely clear and the beads are fully collected. Carefully remove and discard the supernatant.
- ❑ Wash the beads twice by adding 200 µL Wash Buffer, mixing gently, and placing the tube on the magnetic stand. After each wash, discard the supernatant without disturbing the pellet.
- ❑ After the final wash, air-dry the beads briefly (1 minute) on the magnetic stand.
- ❑ To elute the 5′SR–poly(A)+ RNA, add 20 µL of RNase-free molecular biology-grade water to the beads, mix gently by pipetting, and incubate on a thermoblock at 65 °C for 2 minutes with gentle shaking (31 × *g*).
- ❑ Immediately place the tube on the magnetic stand. Once the solution is clear, transfer 20 µL of the supernatant containing the purified 5′SR–poly(A)+ RNA to a pre-cooled RNase-free tube (chilled on ice prior to use).

First-Strand cDNA Synthesis and Amplification

- ❑ Combine 14 µL of purified 5′SR–poly(A)+ RNA with 1 µL of Target RT Primer (poly(T)). Mix gently and incubate at 65 °C for 10 minutes to anneal the primer.
- ❑ Immediately transfer the tube to ice and cool for at least 2 minutes to prevent RNA degradation.
- ❑ Prepare the reverse transcription reaction by adding the remaining components to the primer-RNA mix, according to the composition listed in Table S2.
- ❑ Incubate the complete reaction mixture at 50 °C for 15 minutes for first-strand cDNA synthesis, followed by heat inactivation at 85 °C for 5 minutes.
- ❑ Immediately place the tube on ice to terminate the reaction.
- ❑ Amplify the resulting cDNA by PCR using SeqAmp polymerase, following the reagent composition in Table S3 and the thermal cycling conditions described in Figure S1.

Table S2. Composition of the cDNA synthesis mix

**Reagent**	**Volume (µl)**	**Final concentration**
Primer-RNA mix	15 µl	70.6 %
5x Reaction Mix	4 µl	0.95 x
Maxima Enzyme Mix (200 U)	1 µl	200 U
dNTP	1 µl	0.5 mM
Ribolock (40 U/µL)	0.25µl	10U
**Total**	**21.25 µl**	**-**

Table S3. Composition of the cDNA synthesis reaction

**Reagent**	**Volume (µl)**	**Final concentration**
cDNA mix	20 µl	500 ng
SeqAmp Polymerase	1 µl	1.25 U
2x Buffer	25 µl	1 x
5′ cDNA primer (10 µM)	2 µl	0.4 µM
3′ cDNA primer (10 µM)	2 µl	0.4 µM
**Total**	**50.25 µl**	**-**

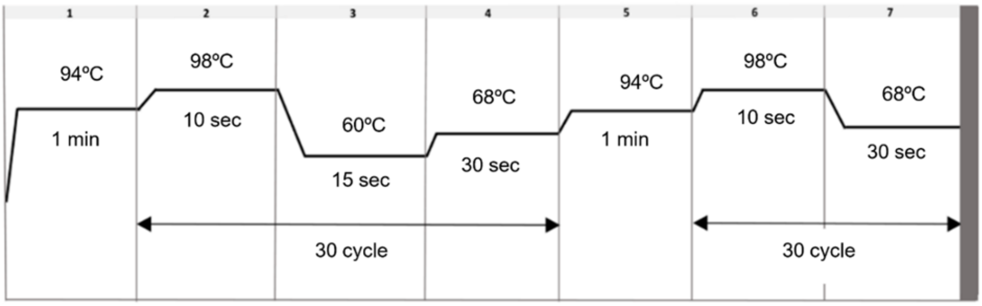

Figure S2. Thermal profile of the cDNA synthesis reaction

Purification of cDNA with AMPure XP Beads and Quality Assessment

- ❑ Equilibrate AMPure XP magnetic beads to room temperature by placing the vial on the bench for at least 30 minutes. Vortex thoroughly before use.
- ❑ Add 80 µL of AMPure XP beads to 50 µL of cDNA sample (beads-to-sample ratio: 1.6:1). Mix thoroughly by pipetting up and down at least 10 times.
- ❑ Incubate the mixture at room temperature for 5 minutes to allow DNA binding to the beads.
- ❑ Place the tube on a magnetic stand until the solution becomes clear (typically ~2 minutes). Carefully remove and discard the supernatant without disturbing the bead pellet.
- ❑ While keeping the tube on the magnetic stand, add 200 µL of freshly prepared 70% ethanol to wash the beads. Incubate for 30 seconds, then remove and discard the ethanol. Repeat the ethanol wash once more (total: 2 washes).
- ❑ After the final wash, allow the beads to air-dry on the magnetic stand until no visible ethanol remains. Avoid overdrying.
- ❑ Elute the purified cDNA by adding 17 µL of RNase-free molecular biology-grade water directly to the beads. Gently pipette to resuspend the pellet fully.
- ❑ Incubate at room temperature for 5 minutes, then place the tube on the magnetic stand. Once the eluate is clear, carefully transfer the entire supernatant to a new RNase-free tube.
- ❑ Assess the concentration and quality of the purified cDNA using the Qubit™ dsDNA High Sensitivity Assay Kit and the Agilent 2100 Bioanalyzer (High Sensitivity DNA Kit), following the manufacturer's protocols.

Note: To prevent bead loss or contamination, the tube must be maintained in a fixed vertical position on the magnetic stand throughout the wash and elution steps.

MmeI Restriction of PCR-Amplified cDNA

- ❑ Prepare the restriction reaction mix according to the composition provided in Table S4. Mix gently by pipetting.
- ❑ Incubate the reaction at 37 °C for 50 minutes.
- ❑ After incubation, allow the reaction mixture to cool gradually to room temperature. Do not place the tube on ice, as rapid cooling may interfere with downstream ligation efficiency.

Table S4. Composition of the Mme I restriction reaction

**Reagent**	**Volume (µl)**	**Final concentration**
PCR Product	15.8 µl	-
10 xNEB 4 Buffer	2 µl	1 x
SAM	0.2 µl	
Mme I (2U/µl)	2 µl	4U
**Total**	**20 µl**	**-**

Duplex Adaptor Annealing and Ligation

- ❑ In a new RNase-free microcentrifuge tube, combine equal volumes (25 µL)of 100 µM dsDNA top strand and 100 µM dsDNA bottom strand adaptors to obtain a final duplex adaptor concentration of 50 µM.
- ❑ Denature the oligonucleotide mixture by incubating at 100 °C for 5 minutes in a thermal cycler or heating block.
- ❑ Gradually cool the mixture to 25 °C at a controlled rate of 0.1 °C/sec to allow proper annealing of the adaptor strands. Do not use snap cooling or ice at this stage.
- ❑ Prepare the duplex adaptor ligation reaction by combining the digested cDNA with the adaptor and ligase mix, according to the composition listed in Table S5.
- ❑ Incubate the ligation reaction at 22 °C for 1 hour to facilitate adaptor ligation.

Table S5. Composition of the duplex adaptor ligation reaction

**Reagent**	**Volume (µl)**	**Final concentration**
Enzymatic digestion product	20 µl	-
Adaptor duplex	2 µl	6.6 µM
T4 DNA ligase (5U/µl)	0.5 µl	2.5 U
10x T4 buffer	3 µl	1x
ddH_2O	4.5 µl	-
**Total**	**30 µl**	**-**

Library Gel Purification and DNA Precipitation

- ❑ Prepare a 4% MetaPhor™ high-resolution agarose gel in 1× TAE buffer. Cast a vertical gel approximately 3 mm thick and allow it to solidify completely.
- ❑ Mix each library sample (25 µL) with 5 µL loading buffer. Load the samples onto the gel alongside 60 bp and 65 bp custom size markers.
- ❑ Run electrophoresis at 150 V for 1.5 hours under cooling conditions (e.g., gel running tank with ice or a cooling plate).
- ❑ Following electrophoresis, transfer the gel into 200 mL of 1× TAE buffer containing 10 mL Midori Green DNA stain. Gently agitate in the dark for 30 minutes.
- ❑ Visualize the gel under UV light at λ = 312 nm. Due to the low concentration of DNA, the libraries typically appear as faint smears.
- ❑ Carefully excise gel fragments corresponding to 60–65 bp based on migrating custom size markers (see Figure S3).
- ❑ Transfer each gel slice into a perforated 0.2 mL tube containing two layers of sterile gauze at the bottom, which is then placed inside a 1.5 mL microcentrifuge tube (figure S4).
- ❑ Centrifuge the assembly at 15,000 × *g* for 10 minutes at room temperature to recover DNA-containing eluate into the lower tube.
- ❑ Transfer the filtrate (~45 µL) to a new RNase-free microcentrifuge tube and add:
    - ▪ 4.5 µL of 3 M sodium acetate (final 1:10 v/v),
    - ▪ 1.0 µL of molecular biology-grade glycogen (20 mg/mL),
    - ▪ 112.5 µL of cold 99% ethanol (2.5:1 ethanol:filtrate, v/v).
- ❑ Mix by inversion and incubate the mixture at –80 °C overnight to precipitate DNA.
- ❑ The next day, centrifuge at 16,000 × *g* for 30 minutes at 4 °C. Carefully decant the supernatant.
- ❑ Wash the DNA pellet with 600 µL of 70% ethanol. Remove the ethanol after 30 seconds by gentle aspiration or pipetting. Do not disturb the pellet.
- ❑ Air-dry the pellet briefly and resuspend in 20 µL of RNase-free molecular biology-grade water. Place the tube at 4 °C for 1 hour.
- ❑ Mix gently by flicking or tapping the tube to ensure complete resuspension. Optionally, centrifuge briefly to collect the dissolved material at the bottom of the tube.

Note: A translucent to white pellet may be visible. Exercise caution during ethanol removal steps to avoid losing the DNA pellet.

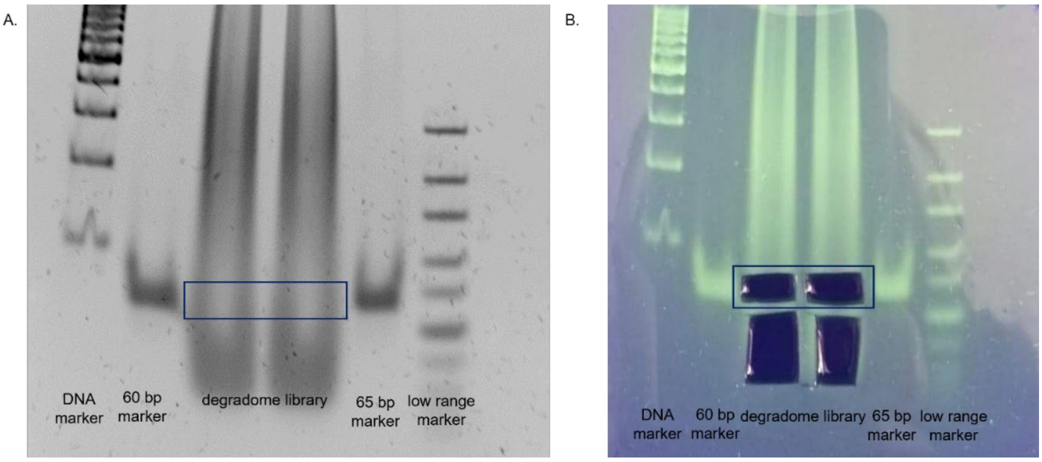

Figure S3. Separation of degradome libraries in 4% high-resolution MetaPhor agarose, Lonza; **A.** Electrophoretic image of the separation of library fragments with 60 bp and 65 bp markers; **B.** image of libraries cut extraction

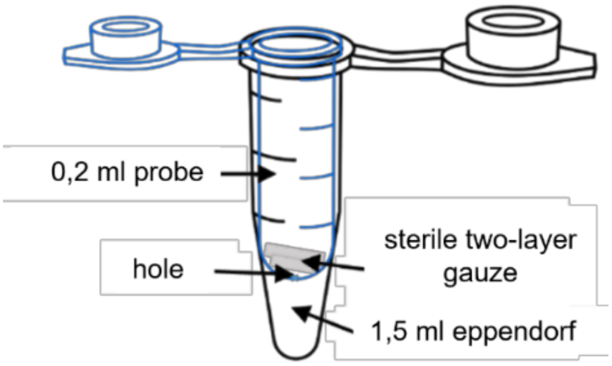

Figure S4. Schematic of a tube for recovery products after electrophoretic separation

PCR Amplification with Illumina-Compatible Primers

- ❑ Prepare the PCR master mix for Illumina-compatible amplification according to the composition provided in Table S6.
- ❑ Add 20 µL of purified gel-extracted library DNA to each 30 µL PCR reaction mix, bringing the final reaction volume to 50 µL.
- ❑ Gently mix the reaction components by pipetting and briefly centrifuge to collect the contents at the bottom of the tube.
- ❑ Perform PCR amplification using the thermal cycling conditions described in Figure S5.
- ❑ Upon completion, purify the amplified libraries using the Pippin Prep system (Sage Science) with a 3% agarose cassette and marker C, following the manufacturer's instructions. Electrophoresis parameters: Start: 100 bp; Stop: 150 bp; Run time: 3 hours.
- ❑ After collection, according to the manufacturers' protocols, assess library quality and concentration using Qubit™ fluorometer (dsDNA High Sensitivity Assay Kit), and Agilent 2100 Bioanalyzer (High Sensitivity DNA Kit).

Table S6. Composition of the PCR amplification reaction with primers compatible with the Illumina platform

Reagent	Volume (µl)	Final concentration
SeqAmp Polymerase	1 µl	1 U
2x Buffer SeqAmp	25 µl	1.25 x
TruSeq Index primer	2 µl	0.625 µM
NebNext SR Primer	2 µl	0.625 µM
**Total**	**30 µl**	**-**

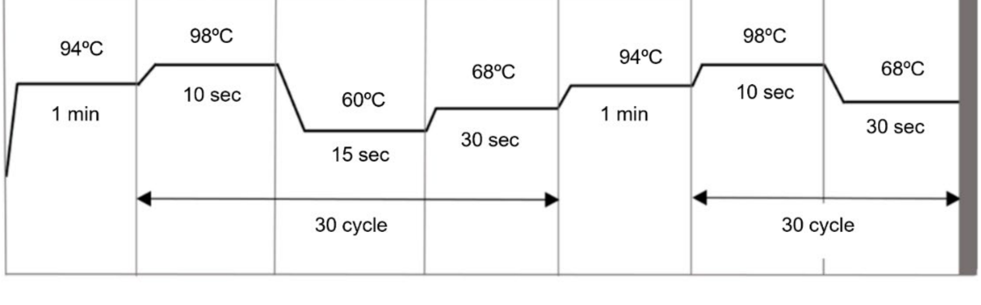

Figure S5. Thermal profile of the cDNA PCR amplification reaction compatible starters for the Illumina platform

Library Denaturation and Sequencing on the Illumina MiSeq Platform

Library Denaturation and Dilution:

- ❑ Combine 2 µL of the 2 nM library with 58.9 µL of molecular biology-grade water in a 1.5 mL tube.
- ❑ Add 5 µL of freshly prepared 0.2 N NaOH, vortex briefly, and centrifuge at 280 × g for 1 minute.
- ❑ Incubate the tube at room temperature for 5 minutes to denature the library.
- ❑ Add 990 µL of pre-chilled HT1 Hybridization Buffer to the denatured library. Do not vortex.
- ❑ The final concentration of the denatured library is 20 pM.

PhiX Control Preparation:

- ❑ Combine 2 µL of 10 nM PhiX control with 3 µL of 10 mM Tris-HCl (pH 8.5).
- ❑ Add 5 µL of 0.2 N NaOH, vortex briefly, and centrifuge at 280 × g for 1 minute.
- ❑ Incubate at room temperature for 5 minutes to denature the PhiX control.
- ❑ Add 990 µL of pre-chilled HT1 Hybridization Buffer. Do not vortex.
- ❑ Mix 375 µL of the 20 pM denatured PhiX with 225 µL of HT1 to obtain a 12.5 pM final concentration. Mix by gentle inversion.

Final Loading:

- ❑ Combine 594 µL of the denatured library with 6 µL of the diluted PhiX control (1% spike-in).
- ❑ Gently mix by pipetting. Do not vortex.
- ❑ Load 600 µL of the prepared library mixture into the designated well of the MiSeq reagent cartridge.

Sequencing:

- ❑ Perform sequencing using the Illumina MiSeq Reagent Kit v3 (50-cycle).
- ❑ Run parameters: Read type: Single-end; Read length: 36 bp; PhiX spike-in: 1%
- ❑ All steps were conducted following the manufacturer's MiSeq System User Guide.
